# Association of Increased Serum ACE Activity with Logical Memory Ability in Type 2 Diabetic Patients with Mild Cognitive Impairment

**DOI:** 10.3389/fnbeh.2016.00239

**Published:** 2016-12-23

**Authors:** Sai Tian, Jing Han, Rong Huang, Wenqing Xia, Jie Sun, Rongrong Cai, Xue Dong, Yanjue Shen, Shaohua Wang

**Affiliations:** ^1^Department of Endocrinology, Affiliated Zhongda Hospital of Southeast UniversityNanjing, China; ^2^Medical School of Southeast UniversityNanjing, China

**Keywords:** angiotensin-converting enzyme, type 2 diabetes mellitus, mild cognitive impairment, polymorphism, memory

## Abstract

**Background:** Angiotensin-converting enzyme (ACE) is involved in the chronic complications of type 2 diabetes mellitus (T2DM) and Alzheimer's disease. This study aimed to assess the pathogenetic roles of ACE and the genetic predisposition of its insertion/deletion (I/D) polymorphism in mild cognitive impairment (MCI) among T2DM patients.

**Methods:** A total of 210 T2DM patients were enrolled. Among these patients, 116 satisfied the MCI diagnostic criteria and 94 exhibited healthy cognition. The cognitive functions of the patients were extensively assessed. The serum level and activity of ACE were measured via enzyme-linked immunosorbent assay and ultraviolet spectrophotography. The single-nucleotide polymorphisms of I/D gene of ACE were analyzed.

**Results:** The serum level and activity of ACE in diabetic MCI patients (*p* = 0.022 and *p* = 0.008, respectively) were both significantly higher than those in the healthy controls. A significant negative correlation was found between their ACE activity and logical memory test score (LMT) (*p* = 0.002). Multiple stepwise regression iterated the negative correlation between ACE activity and LMT score (*p* = 0.035). Although no significant difference was found in the genotype or allele distribution of ACE I/D polymorphism between the groups, the serum levels and activity of ACE were higher in the DD group than in the ID and II groups (*p* < 0.05).

**Conclusions:** Serum ACE activity could better predict logical memory in T2DM patients than ACE level. Further investigations on a large population size are necessary to test whether the D-allele of the ACE gene polymorphism is susceptible to memory deterioration.

## Introduction

Several epidemiological studies have shown that type 2 diabetes mellitus (T2DM) may exert influence on the prevalence of mild cognitive impairment (MCI) (Luchsinger et al., 2007), which is a transitional stage between normal aging and dementia. Patients with diabetes have higher risks of MCI, particularly memory ability, than those without diabetes (Cheng et al., 2012). Several explanations for the link between diabetes and cognitive dysfunction have been provided, including chronic hyperglycemia (Strachan et al., 2011), recurrent hypoglycemia (Muratli et al., 2015), insulin deficiency (Ma et al., 2015), reduced cerebral blood flow(Strachan et al., 2011; Glodzik et al., 2013; Nealon et al., 2016), amyloid β (Aβ) deposition (Yang and Song, 2013), tau protein hyperphosphorylation (McCrimmon et al., 2012) and so on. However, the exact mechanism remains unclear.

Angiotensin-converting enzyme (ACE), which is one of the key components of the renin–angiotensin system (RAS), is a zinc metallopeptidase that converts angiotensin I to angiotensin II (Ang II) (Tiret et al., 1998; Dhar et al., 2012). All organs of the body are now recognized to have their own local paracrine-like RAS with organ-specific functions (Kehoe et al., 2009). The actions of angiotensin II within the central nervous system are of increasing interest in the context of Alzheimer's disease (AD). Ang II may lead to reduction in cerebral blood flow because of vasoconstriction (Inaba et al., 2009) or oxidative stress (Tota et al., 2012). Besides, angiotensin II inhibits the release of acetylcholine from the human temporal cortex (Barnes et al., 1990) and has a pro-inflammatory effect (Kehoe, 2009). Findings from *in vitro* studies and animal models have shown that ACE may play an important role in the metabolism of Aβ (Hu et al., 2001; Oba et al., 2005). The level and activity of ACE within the cerebral cortex are generally elevated in AD patients (Arregui et al., 1982; Barnes et al., 1991; He et al., 2006; Miners et al., 2008). Several studies have found that insertion/deletion (I/D) polymorphism in the ACE gene is associated with ACE levels and activity (Rigat et al., 1990) and can be associated with AD risk, whereas the D allele exhibits different results (Elkins et al., 2004; Lehmann et al., 2005; Helbecque et al., 2009).

The association between T2DM and AD has been described in various clinical studies (de la Monte and Wands, 2008; Moreira et al., 2013). High levels of angiotensin II have been reported to possibly play a key role in glucose and insulin regulation and may increase the risk of diabetes (Zhou et al., 2012). The association of the ACE gene with insulin resistance and inflammatory factors has been reported (Huang et al., 1998; Stephens et al., 2005). This study was designed to assess the association between serum ACE level/activity and cognition performance. In addition, it aimed to determine whether I/D polymorphism in the ACE gene was associated with diabetic MCI. The results of our study provide additional insights into the pathogenesis of T2DM with MCI and an early therapeutic strategy for dementia.

## Materials and methods

### Subjects and study design

The study was conducted in the Department of Endocrinology of the Affiliated Zhongda Hospital of Southeast University. All the participants were Chinese Han and provided written informed consents according to a protocol approved by the Research Ethics Committee of the Affiliated Zhongda Hospital of Southeast University.

We recruited 210 (119 men and 91 women, aged 50–75 years) hospitalized patients who satisfied the diagnostic criteria of T2DM. Among these individuals, 116 were diabetic patients with MCI and 94 were diabetic patients with healthy cognition. The control group was composed of diabetic patients with healthy cognition. These patients were diagnosed with T2DM according to the World Health Organization 1999 criteria (Alberti and Zimmet, 1998). All the MCI patients satisfied the diagnostic criteria proposed by the MCI Working Group of the European Consortium on Alzheimer's Disease in 2006: (1) cognitive complaints from patients or their families; (2) decline in cognitive functioning in the past year relative to previous abilities, as reported by the patient or an informant, with a Clinical Dementia Rating (CDR) score of 0.5; (3) cognitive disorders as evidenced by clinical evaluation (impairment in memory or in another cognitive domain); (4) the absence of major repercussions in daily life (however, a patient may report difficulties in performing complex day-to-day activities); and (5) the absence of dementia (Portet et al., 2006). The following exclusion criteria were considered in this study: diabetic ketoacidosis, hyperosmolar nonketotic diabetic coma, severe hypoglycemia, acute cardiovascular or cerebrovascular accident, history of stroke (Hachinski score ≥ 4), head injury, alcoholism, Parkinson's disease, epilepsy, major depression or other physical and mental illnesses, major medical illness (e.g., cancer, anemia, and serious infection), and severe visual or hearing loss (e.g., diabetic retinopathy, glaucoma, cataract, otitis media, deafness).

### Clinical data collection

Demographic characteristics, including age, gender, education levels, occupation, and contact information, were collected. Medical histories (including hypertension, coronary heart disease, and cerebral infarction) and physical measurements (including blood pressure, weight, and height) were obtained using a standard balance beam scale. Medication history, including insulin, Angiotensin Converting Enzyme Inhibitors (ACEI), and Angiotensin Receptor Blockers (ARB), were collected. Body mass index (BMI) was defined as the body weight of an individual divided by the square of his/her height [body weight (kg)/body height (m^2^)]. Patients with a systolic blood pressure of ≥ 140 mmHg and a diastolic blood pressure of ≥ 90 mmHg were considered hypertensive. Blood samples were obtained to determine the levels of glycosylated hemoglobin (HbA1c, %), triglyceride (TG), total cholesterol (TC), low-density lipoprotein cholesterol (LDL-C), and high-density lipoprotein cholesterol (HDL-C). The central laboratory of Zhongda Hospital implements internal and external quality control procedures as directed by the Chinese Laboratory Quality Control.

### Neuropsychological tests

A battery of neuropsychological tests, including the Montreal Cognitive Assessment (MoCA) (Gil et al., 2015), digit span test (DST) (Leung et al., 2011), verbal fluency test (VFT), clock drawing test (CDT) (Yoo and Lee, 2016), word similarity test (ST), auditory verbal learning test (AVLT) (Hong et al., 2012), logical memory test (LMT) (Chapman et al., 2016), Stroop color–word test (SCWT) (Balota et al., 2010), and trail making tests A and B (TMT-A and TMT-B) (Albert et al., 2001), were performed to evaluate the cognitive functions, such as semantic memory, attention, psychomotor speed, executive function, and visuospatial skills, of each subject. Hachinski ischemic score, clinical dementia rating (CDR), activity of daily living scale (ADL), and self-rating depression scale (SDS) were also obtained. Approximately 50 min were used to complete the tests in a fixed order. An experienced neuropsychiatrist facilitated the tests, and all the subjects were not informed of the study design.

### Measurement of serum ACE level and activity

Blood samples (2 mL) were collected between 6:30 a.m. and 7:00 a.m. in fasted state via venipuncture in anticoagulant-free tubes. The blood samples were centrifuged at 100 × g for 15 min. Serum was collected and stored at −80°C until use. Fasting serum ACE concentrations were measured using enzyme-linked immunosorbent assay kits [R&D Systems, Minneapolis, MN, USA] according to the instructions of the manufacturer. The serum ACE levels of the subjects were measured during the same day to minimize assay variance. Fasting serum ACE activity was determined under ultraviolet spectrophotography using a Hitachi 7170 fully automatic biochemical analyzer in accordance with the instructions of the manufacturer indicated in the ACE activity kit (Zhejiang Kuake Bioscience Technology Co., Ltd.). Serum ACE activity was measured in all the subjects on the same day to minimize assay variance. The intra-assay coefficient of variation was less than 6%.

### Genotyping of ACE I/D polymorphism

Genomic DNA was extracted from 2 mL EDTA anticoagulated-venous blood using a DNA Purification Kit (Gentra, Minnesota, USA) according to the recommendations of the manufacturer. Polymerase chain reaction (PCR)−restriction fragment length polymorphism was conducted to genotype the DNA sequence variants of the ACE gene I/D. The primer sequences were forward primer (5′-GGACTCTGTAAGCCACTG-3′) and reverse primer (5′-CTCCCATGCCCATAAC-3′). PCR was conducted in 30 μL reaction mixtures with 20.8 μL ddH_2_O, 3.0 μL 10 × PCR buffer, 60 ng DNA, 10 pmol primer forward, 10 pmol primer reverse, and 2 μL dNTP. The amplification conditions were initiated at 96°C for 5 min, followed by 30 cycles of denaturation at 96°C for 20 s, annealing at 55°C for 20 s, extension at 72°C for 30 s, and a final extension step at 72°C for 10 min. The PCR products were analyzed using 2% agarose gel electrophoresis and ethidium bromide staining to identify three patterns: I/I (490 bp band), D/D (190 bp band), and I/D (both 490 and 190 bp bands).

### Statistical analysis

Data were reported as mean ± standard error of mean (SEM), median (interquartile range), or percentage, as appropriate. Student's *t*-test and analysis of variance (ANOVA) were performed to compare the normally distributed variables. Nonparametric Mann–Whitney U and Kruskal–Wallis tests were conducted to compare the asymmetrically distributed variables. Chi-squared (χ^2^) test was used to compare the qualitative variables. This test was also conducted to evaluate the distribution of genotypes and allele frequencies as well as to determine the deviations from the Hardy–Weinberg equilibrium (Santiago Rodriguez, Tom R. Gaunt, and Ian N. M. Day, Hardy–Weinberg Equilibrium Testing of Biological Ascertainment for Mendelian Randomization Studies). The correlation between the neuropsychological test scores and serum ACE level or activity was examined using Pearson's or Spearman's correlation. Multiple stepwise regression analysis was conducted to investigate the relationship of cognitive performances with demographic characteristics, clinical characteristics, and serum ACE level or activity. Statistical analysis was conducted using SPSS 19.0 (SPSS Inc., Chicago, IL). A two-sided *p* < 0.05 was defined as statistically significant.

## Results

### Demographic characteristics, clinical characteristics, and cognitive performances

The demographic characteristics, clinical characteristics, and neuropsychological test scores of the participants are listed in Table 1. The MCI group and the control group were well matched in terms of age, gender distribution, educational level, smoking history, drinking history, BMI, hypertension prevalence, diabetes duration, insulin use, and ACEI or ARB use (*p* > 0.05). No significant difference was found in TG, TC, LDL-C, HDL-C, ApoA1, and ApoB levels between the two groups. T2DM patients with MCI had elevated fasting blood glucose (FBG) and HbAlc and lower fasting C-peptide than the control group (*p* < 0.05). The neuropsychological test scores of the MCI patients, except for the correct numbers of Card A, were significantly lower than those of the control subjects (*p* < 0.01).

**Table 1:** **Demographic characteristics, clinical characteristics, cognitive performances, serum ACE levels and ACE activity**.

**Characteristic**	**MCI group (*n* = 116)**	**Control group (*n* = 94)**	***p*****-value**
Age (years)	60.88 ± 0.59	59.33 ± 0.76	0.103^a^
Female, n (%)	56.00 (48.30)	35.00 (37.20)	0.108^c^
Education Levels (years)	9.00 (9.00–12.00)	9.00 (9.00–12.00)	0.130^b^
Smoking, n (%)	48.00 (41.40)	42.00 (44.70)	0.631^c^
Drinking, n (%)	33.00 (28.40)	22.00 (23.40)	0.408^c^
BMI (kg/m^2^)	25.25 ± 0.33	25.21 ± 0.35	0.928^a^
Hypertension, n (%)	74.00 (63.80)	50.00 (53.20)	0.120^c^
Hypertension duration (years)	6.00 (0.00–10.75)	3.50 (0.00–10.00)	0.189^b^
Systolic pressure (mmHg)	140.00 (125.00–150.00)	132.00 (124.00–141.25)	0.068^b^
Diastolic pressure (mmHg)	80.00 (75.00–90.00)	80.00 (75.00–90.00)	0.423^b^
Diabetes duration (years)	10.00 (5.25–15.00)	8.00 (5.00–13.00)	0.228^b^
Use of insulin, n (%)	79.00 (68.10)	54.00 (57.40)	0.111^c^
Use of ACEI OR ARB, n (%)	25.00 (21.60)	26.00 (27.70)	0.305^c^
HbA1c (%)	9.59 ± 0.23	8.84 ± 0.24	0.026^a^^*^
FBG (mmol/L)	8.30 ± 0.24	7.61 ± 0.23	0.041^a^^*^
PBG (mmol/L)	14.67 ± 0.32	13.78 ± 0.39	0.076^a^
Glucose fluctuation (mmol/L)	6.80 (5.08–9.53)	6.25 (4.28–9.13)	0.150^b^
FCP (nmol/L)	1.39 (1.03–1.85)	1.77 (1.32–2.53)	<0.001^b^^*^
Triglyceride (mmol/L)	1.33 (1.04–1.86)	1.50 (1.24–1.94)	0.063^b^
Total cholesterol (mmol/L)	4.88 ± 0.11	4.80 ± 0.12	0.609^a^
LDL-cholesterol (mmol/L)	2.99 ± 0.08	2.97 ± 0.09	0.873^a^
HDL-cholesterol (mmol/L)	1.18 ± 0.03	1.19 ± 0.02	0.881^a^
ApoA1 (g/L)	1.06 ± 0.02	1.11 ± 0.02	0.162^a^
ApoB (g/L)	0.87 ± 0.02	0.86 ± 0.02	0.733^a^
**COGNITION TEST LEVELS**			
MoCA	21.00 (18.00–23.00)	27.00 (26.00–28.00)	<0.001^b^^*^
DST	11.00 (8.00–12.00)	12.00 (11.00–14.00)	<0.001^b^^*^
VFT	14.70 ± 0.35	17.20 ± 0.42	<0.001^a^^*^
CDT	3.00 (2.00–4.00)	4.00 (4.00–4.00)	<0.001^b^^*^
ST	7.00 (5.00–9.00)	9.00 (8.00–10.00)	<0.001^b^^*^
TMT-A	81.00 (61.50–108.50)	59.00 (47.75–71.00)	<0.001^b^^*^
TMT-B	197.00 (150.00–263.75)	145 (114–180)	<0.001^b^^*^
SCWT A time	35.69 ± 0.86	28.41 ± 0.62	<0.001^a^^*^
SCWT A number	50.00 (49.00–50.00)	50.00 (50.00–50.00)	0.165^b^
SCWT B time	59.43 ± 1.03	42.56 ± 0.92	<0.001^a^^*^
SCWT B number	48.00 (46.00–49.00)	50.00 (49.00–50.00)	<0.001^b^^*^
SCWT C time	117.38 ± 2.37	84.02 ± 1.45	<0.001^a^^*^
SCWT C number	44.50 (42.00–46.00)	47.00 (45.00–50.00)	<0.001^b^^*^
AVLT immediate recall	15.47 ± 0.39	20.33 ± 0.45	<0.001^a^^*^
AVLT delayed recall	4.00 (3.00–6.00)	7.00 (5.00–9.00)	<0.001^b^^*^
LMT	4.00 (2.00–8.00)	10.00 (7.75–13.00)	<0.001^b^^*^
ACE level (ng/mL)	200.16 ± 8.96	171.14 ± 8.48	0.022^a^^*^
ACE activity (u/L)	47.45 (26.13–86.68)	33.15 (23.25–52.43)	0.008^b^^*^

* *Significance, p <0.05*.

*Data are presented as n (%), mean ± SEM, or median (interquartile range) as appropriate*.

a *Student's t test for comparison of normally distributed quantitative variables between MCI group and control group*.

b *Mann-Whitney U test for comparison of asymmetrically distributed quantitative variables between MCI group and control group*.

c *χ^2^ test for comparison of qualitative variables between MCI group and control group. Abbreviations: MCI, mild cognitive impairment; BMI, body mass index; HbAlc, glycosylated hemoglobin; FBG, fasting blood glucose; PBG, postprandial blood glucose; FCP, Fasting c-peptide; LDL, low-density lipoprotein; HDL, high-density lipoprotein; MoCA, Montreal Cognitive Assessment; DST, Digit Span Test; VFT, Verbal Fluency Test; CDT, Clock Drawing Test; ST, Similarities test; TMT-A, Trail Making Test-A;TMT-B, Trail Making Test-B; SCWT, Stroop Color Word Test; AVLT, Auditory Verbal Learning Test; LMT, Logical Memory Test; ACE, angiotensin-converting enzyme*.

### Serum ACE levels and activity between the MCI and the control group

The MCI group exhibited markedly higher serum ACE level and activity than the control group [200.16 ± 8.96 vs. 171.14 ± 8.48, *p* = 0.022; 47.45 (26.13–86.68) vs. 33.15 (23.25–52.43), *p* = 0.008, respectively; Table 1].

### Respective relationship between cognitive performances and serum ACE level and activity

Significant differences were found in serum ACE level, ACE activity, and cognitive performances between the two groups. The respective correlations between cognitive performances and serum ACE level/activity were analyzed. Pearson's or Spearman's correlation showed significantly negative correlations between MoCA and LMT scores and ACE activity in the MCI subgroup (*r* = −0.242, *p* = 0.009; *r* = −0.286, and *p* = 0.002, respectively; Table 2). Besides, there was a significantly positive correlation between VFT and serum ACE level (*r* = 0.195, *p* = 0.036; Table 2). By contrast, no significant correlation was found between DST, CDT, ST, AVLT, or SCWT scores and serum ACE level/activity (*p* > 0.05; Table 2). When LMT score was considered as a dependent variable, and age, educational level, diabetes duration, hypertension duration, FBG, blood lipid levels, and serum ACE activity were considered as independent variables in the multiple stepwise regression analysis, the results indicated that the LMT score was significantly associated with educational level, TC, and serum ACE activity (β = 0.265, *p* = 0.003; β = −0.205, *p* = 0.020; β = −0.186, *p* = 0.035, respectively; Table 3).

**Table 2:** **Relationships between serum ACE level, ACE activity and cognitive performances in T2DM patients with MCI**.

	**Serum ACE level**		**Serum ACE activity**	
	**r**	***p*****-value**	**r**	***p*****-value**
MoCA	−0.145	0.122^b^	−0.242	0.009^a^^*^
DST	0.003	0.973^b^	−0.056	0.554^a^
VFT	0.195	0.036^a^^*^	−0.081	0.390^a^
CDT	−0.092	0.326^b^	−0.026	0.783^a^
ST	0.104	0.268^a^	−0.054	0.562^a^
TMT-A	0.033	0.723^b^	−0.003	0.974^a^
TMT-B	0.039	0.681^b^	0.029	0.758^a^
SCWT-A Time	0.048	0.608^a^	0.078	0.404^a^
SCWT-A Number	−0.044	0.637^b^	−0.031	0.737^a^
SCWT-B Time	0.006	0.950^a^	0.051	0.590^a^
SCWT-B Number	0.117	0.211^b^	−0.061	0.515^a^
SCWT-C Time	0.057	0.546^b^	0.064	0.495^a^
SCWT-C Number	0.045	0.628^a^	−0.065	0.486^a^
AVLT immediate recall	−0.037	0.693^a^	−0.095	0.310^a^
AVLT delayed recall	−0.086	0.361^b^	−0.148	0.113^a^
LMT	−0.104	0.268^b^	−0.286	0.002^a^^*^

* *Significance, p <0.05*.

a *Pearson correlation*.

b *Spearman correlation. Abbreviations: MCI, mild cognitive impairment; MoCA, Montreal Cognitive Assessment; DST, Digit Span Test; VFT, Verbal Fluency Test; CDT, Clock Drawing Test; ST, Similarities test; TMT-A, Trail Making Test-A;TMT-B, Trail Making Test-B; SCWT, Stroop Color Word Test; AVLT, Auditory Verbal Learning Test; LMT, Logical Memory Test; ACE, angiotensin-converting enzyme*.

**Table 3:** **Multiple linear regression analysis of factors associated with LMT scores in T2DM patients with MCI**.

	**Standardized β**	**95%CI**		**P**
		**Lower**	**Upper**	
Education level	0.265	0.134	0.629	0.003^*^
TC	−0.205	−1.182	−0.105	0.020^*^
Serum ACE activity	−0.186	−0.022	−0.001	0.035^*^

* *Significance, p <0.05*.

*Abbreviations: LMT, Logical Memory Test; TC, Total cholesterol; ACE, angiotensin-converting enzyme*.

### Distributions of ACE genotype and allele frequencies between groups

The ACE genotype and allele frequencies of the MCI patients and the control subjects are shown in Table 4. The distribution of the ACE genotypes was consistent with the Hardy–Weinberg equilibrium in the MCI group (χ^2^ = 3.45, *df* = 1, *p* > 0.05) and the control group (χ^2^ = 3.38, *df* = 1, *p* > 0.05). No significant difference was found in the distributions of the ACE genotypes (χ^2^ = 0.038, *df* = 2, *p* = 0.981) and allele frequencies (χ^2^ = 0.028, *df* = 1, *p* = 0.867) between the MCI group and the control group.

**Table 4:** **Distributions of ACE genotype and allele frequencies between groups**.

	**Genotype, n (%)**				**Allele, n (%)**		
**Group**	**DD**	**ID**	**II**	***p*****-value^a^**	**D**	**I**	***p*****-value^a^**
MCI	50 (43.10)	45 (38.80)	21 (18.10)	0.981	145 (62.50)	87 (37.50)	0.867
Control	40 (42.60)	36 (38.30)	18 (19.10)		116 (61.70)	72 (38.30)	

*Data are presented as n (%)*.

a *χ^2^ test for comparison of genotype and allele frequencies between MCI group and control group. Abbreviations: MCI, mild cognitive impairment; ACE, angiotensin-converting enzyme*.

### Comparison of serum ACE level, ACE activity, and cognitive performances between genotypic subgroups

**Figure 1:**
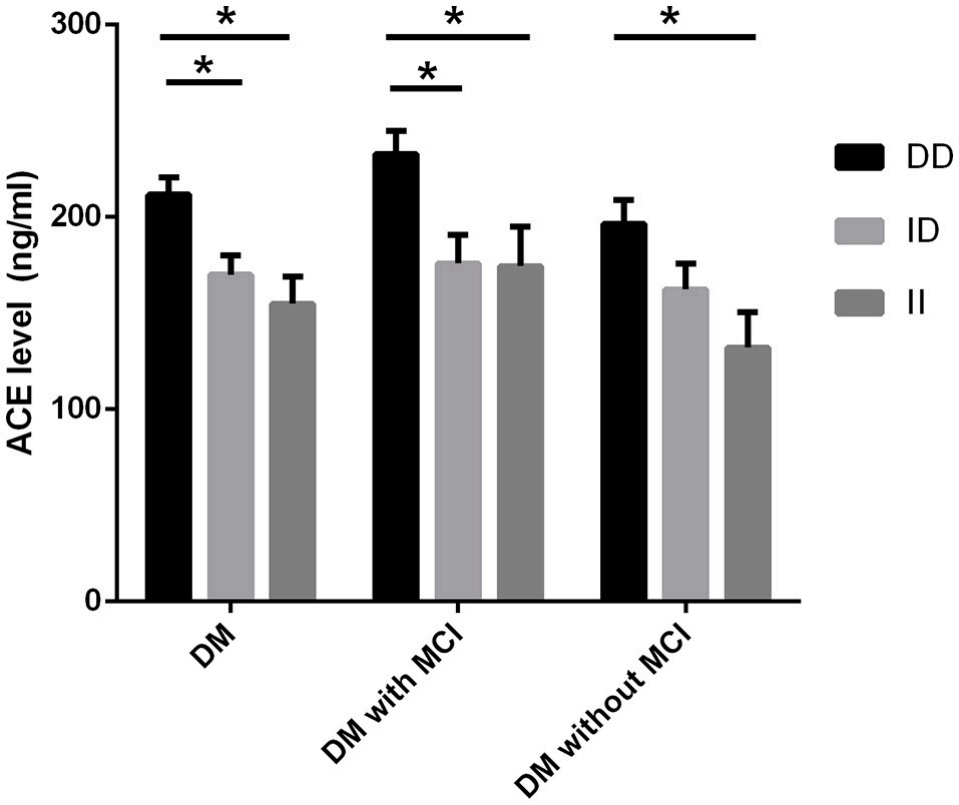
**Comparison of serum ACE level between genotypic subgroups**. Analysis of variance (ANOVA) is used for comparison of serum level of ACE between different genotypes. In the MCI group, further statistical analysis showed that serum ACE level was significantly greater in the DD group than in the ID and II groups. The error bars represent the SEM. ^*^p < 0.05.

**Figure 2:**
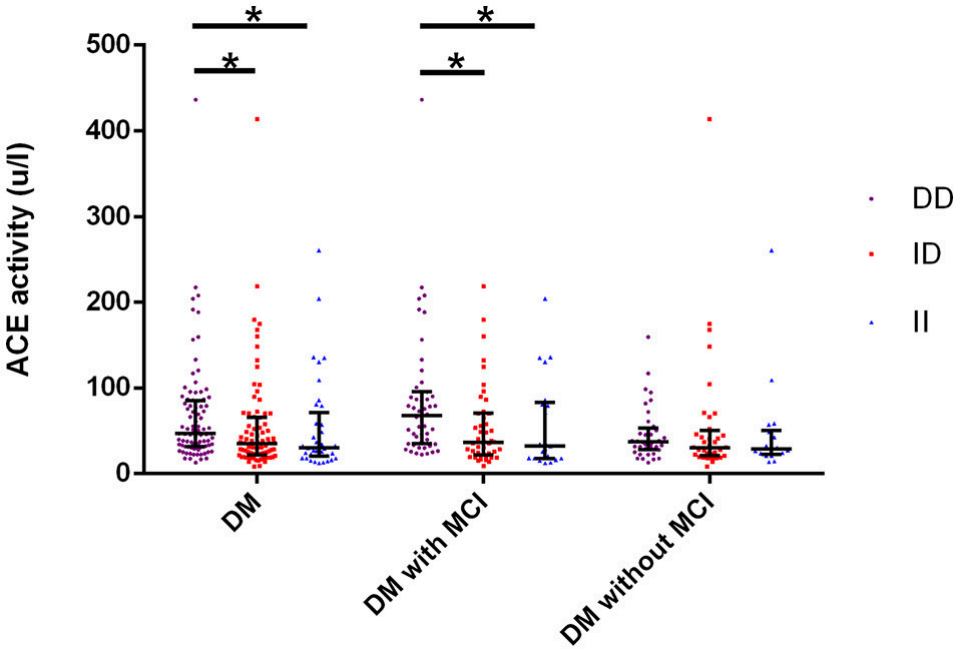
**Comparison of serum ACE activity between genotypic subgroups**. Kruskal-wallis H(k) test is used for comparison of serum activity of ACE between different genotypes. In the MCI group, further statistical analysis showed that serum ACE activity was significantly greater in the DD group than in the ID and II groups. The super and lower horizontal line represent the 25th- and 75th- percentile values (P25 and P75). The median line represents the 50th- percentile value (median, P50). ^*^p < 0.05.

Serum ACE levels were significantly different among the three genotypic subgroups (DD, ID, and II) in the DM, MCI, and healthy-cognition control groups (*p* < 0.001, *p* = 0.006, *p* = 0.014, respectively; Table 5). Serum ACE activity was significantly different among the three genotypic subgroups (DD, ID, and II) in the DM group and the MCI group (*p* = 0.003 and *p* = 0.005, respectively; Table 6). In the MCI group, further statistical analysis showed that serum ACE level and activity were both significantly greater in the DD group than in the ID and II groups (*p* < 0.05, Figures 1, 2). In the healthy-cognition control group, the serum ACE level of the DD subgroup was only different from that of the II genotypic subgroup (*p* < 0.05, Figure 1). However, serum ACE activity was not significantly different in the three genotypic subgroups in the healthy-cognition control group (*p* > 0.05, Figure 2). The neuropsychological test scores were also not significantly different in the genotypic subgroups in the MCI and control subjects (*p* > 0.05, Table 7).

**Table 5:** **Comparison of serum ACE level between genotypic subgroups**.

**Group**	**Genotype**			***p*****-value^a^**
	**DD**	**ID**	**II**	
DM	216.66 ± 8.87	169.93 ± 10.16	154.91 ± 14.25	<0.001
DM with MCI	232.75 ± 12.07	175.94 ± 14.93	174.45 ± 20.69	0.006
DM without MCI	196.55 ± 12.52	162.42 ± 13.32	132.11 ± 18.42	0.014

*Data are presented as mean ± SEM*.

a *Analysis of variance (ANOVA) for comparison of serum level of ACE between different genotypes. Abbreviations: MCI, mild cognitive impairment; ACE, angiotensin-converting enzyme*.

**Table 6:** **Comparison of serum ACE activity between genotypic subgroups**.

**Group**	**Genotype**			***p*****-value^a^**
	**DD**	**ID**	**II**	
DM	46.70 (31.50–85.48)	35.20 (22.05–65.80)	30.20 (20.60–71.50)	0.003
DM with MCI	68.05 (34.83–95.60)	36.70 (22.15–70.35)	32.10 (17.95–83.45)	0.005
DM without MCI	37.20 (28.30–53.25)	30.55 (21.55–50.80)	28.85 (22.40–50.75)	0.407

*Data are presented as median (interquartile range)*.

a *Kruskal-wallis H(k) test for comparison of serum activity of ACE between different genotypes. Abbreviations: MCI, mild cognitive impairment; ACE, angiotensin-converting enzyme*.

**Table 7:** **Comparison of cognitive performances between genotypic subgroups**.

**Cognitive performances**	**MCI group (*n* = 116)**				**Control group (*n* = 94)**			
	**DD (*n* = 50)**	**ID (*n* = 45)**	**II (*n* = 21)**	***p*****-value**	**DD (*n* = 40)**	**ID (*n* = 36)**	**II (*n* = 18)**	***p*****-value**
MoCA	21.00 (19.00–23.00)	21.00 (17.50–24.00)	19.00 (16.00–23.50)	0.551^b^	27.00 (26.00–27.00)	26.50 (26.00–28.00)	27.00 (26.00–28.00)	0.557^b^
DST	10.50 (8.00–12.00)	10.00 (8.00–11.00)	11.00 (9.00–12.00)	0.426^b^	12.20 ± 0.29	12.31 ± 0.37	11.72 ± 0.37	0.572^a^
CDT	3.00 (2.00–4.00)	3.00 (2.00–4.00)	3.00 (2.00–4.00)	0.786^b^	4.00 (4.00–4.00)	4.00 (3.25–4.00)	4.00 (3.00–4.00)	0.590^b^
VFT	15.20 ± 0.60	14.07 ± 0.48	14.86 ± 0.75	0.335^a^	17.65 ± 0.70	17.58 ± 0.60	15.44 ± 0.91	0.126^a^
ST	7.36 ± 0.35	7.18 ± 0.34	6.71 ± 0.74	0.634^a^	9.00 (8.00–10.00)	9.00 (8.25–10.00)	8.00 (8.00–11.00)	0.723^b^
TMT–A	81.00 (64.00–98.75)	73.00 (58.00–108.00)	87.00 (57.50–133.00)	0.875^b^	59.03 ± 2.68	62.28 ± 3.08	67.00 ± 6.53	0.369^a^
TMT–B	196.00 (152.00–242.00)	193.00 (141.00–282.50)	206.00 (138.00–369.50)	0.649^b^	155.78 ± 9.03	139.36 ± 7.32	173.56 ± 13.13	0.073^a^
SCWT A number	50.00 (49.00–50.00)	50.00 (49.00–50.00)	50.00 (49.50–50.00)	0.956^b^	50.00 (50.00–50.00)	50.00 (50.00–50.00)	50.00 (49.00–50.00)	0.412^b^
SCWT B number	48.00 (46.00–49.00)	47.00 (46.00–49.00)	48.00 (46.00–50.00)	0.661^b^	50.00 (49.25–50.00)	50.00 (49.00–50.00)	50.00 (49.00–50.00)	0.751^b^
SCWT C number	43.92 ± 0.44	43.91 ± 0.54	44.52 ± 0.61	0.742^a^	47.50 (46.00–50.00)	47.50 (44.25–49.00)	45.00 (43.75–49.25)	0.152^b^
SCWT A time	35.28 ± 1.42	35.38 ± 1.35	37.33 ± 1.72	0.671^a^	27.93 ± 0.98	28.56 ± 1.00	29.22 ± 1.40	0.741^a^
SCWT B time	58.90 ± 1.54	59.36 ± 1.84	60.86 ± 1.99	0.797^a^	41.75 ± 1.24	43.42 ± 1.77	42.67 ± 1.83	0.721^a^
SCWT C time	113.00 (102.00–127.00)	118.00 (99.00–126.00)	118.00 (103.50–127.50)	0.941^b^	81.30 ± 1.89	84.86 ± 2.82	88.39 ± 2.66	0.187^a^
AVLT immediate recall	16.18 ± 0.53	15.31 ± 0.66	14.14 ± 0.94	0.159^a^	20.53 ± 0.65	20.61 ± 0.82	19.33 ± 0.87	0.561^a^
AVLT delayed recall	4.00 (3.00–6.00)	4.00 (3.00–7.00)	4.00 (2.00–5.00)	0.704^b^	7.00 (6.00–7.75)	6.00 (5.00–9.00)	7.00 (5.00–9.00)	0.774^b^
LMT	4.00 (2.00–7.00)	3.00 (1.00–9.50)	3.00 (1.50–8.00)	0.983^b^	9.45 ± 0.61	10.58 ± 0.55	9.22 ± 1.07	0.325^a^

*Data are presented as mean ± SEM, or median (interquartile range) as appropriate*.

a *Analysis of variance (ANOVA) for comparison of normally distributed quantitative variables between genotypic subgroups in MCI group and control group*.

b *Kruskal-Wallis test for comparison of asymmetrically distributed quantitative variables between genotypic subgroups in MCI group and control group. Abbreviations: MCI, mild cognitive impairment; MoCA, Montreal Cognitive Assessment; DST, Digit Span Test; VFT, Verbal Fluency Test; CDT, Clock Drawing Test; ST, Similarities test; TMT-A, Trail Making Test-A;TMT-B, Trail Making Test-B; SCWT, Stroop Color Word Test; AVLT, Auditory Verbal Learning Test; LMT, Logical Memory Test*.

## Discussion

To find the susceptible genotype and peripheral blood biomarkers of cognitive deficits in diabetic patients, our previous studies focused on the insulin-related pathway (Huang et al., 2015) and the glucose toxicity pathway (Wang et al., 2016) during the early and mild stages of cognitive impairment among diabetic patients. ACE is one of the key components of RAS, and it converts angiotensin I to angiotensin II (Tiret et al., 1998; Dhar et al., 2012). Ang II may lead to reduction in cerebral blood flow because of vasoconstriction (Inaba et al., 2009) or oxidative stress (Tota et al., 2012). Besides, angiotensin II inhibits the release of acetylcholine from the human temporal cortex (Barnes et al., 1990) and has a pro-inflammatory effect (Kehoe, 2009). Cholinergic system in the hippocampus plays an important role in memory formation and retrieval (Popik et al., 1994). Animal and *in vitro* studies both have shown that ACE plays an important role in the metabolism of Aβ (Hu et al., 2001; Oba et al., 2005). High levels of angiotensin II have been reported to possibly play a key role in regulating glucose and insulin and may increase the risk of diabetes (Zhou et al., 2012). ACE is likely to play a role in diabetic cognitive impairment via vascular factors (Pernomian et al., 2014). Therefore, this study aims to investigate the roles of ACE in diabetic cognitive impairment and the relationship of its polymorphism with the disease.

The most striking finding is that both serum ACE level and activity have been determined to be significantly higher in diabetic MCI patients than in the healthy-cognition controls. The serum ACE activity of diabetic MCI patients was negatively correlated with their LMT scores, which represented logical memory, and was iterated via multiple stepwise regressions. ACE serum level did not correlate with any of the behavioral scores. The ACE activity of the DD genotype was significantly higher than that of ID and II genotypes in diabetic patients with MCI, which is consistent with previous results (Rigat et al., 1990; Lehmann et al., 2005; Biller et al., 2006; Zhang et al., 2010, 2011). Nevertheless, no significant association between ACE I/D polymorphism and MCI was observed in T2DM patients.

Our study found that serum ACE level and activity were both increased in T2DM patients with MCI, which was consistent with previous results in rats with early-onset diabetes (Yamaleyeva et al., 2012). We noticed that our MCI patients had higher levels of HbA1C, which indicated poor glucose control. Hyperglycemia has been shown to increase serum ACE level and activity (Härdtner et al., 2013). In addition, elevated ACE activity could reduce the release of neprilysin (NEP) which acts as an Aβ-degrading enzyme in the brain (Carson and Turner, 2002). This finding can probably be applied to diabetic patients with cognitive impairment. Aβ accumulation is regarded as a predictive factor of cognitive deficits among diabetic patients (Sato et al., 2010) and is detected during their early stage of cognitive impairment (Yang and Song, 2013). However, the accurate content of Aβ in the brain is extremely difficult to be detected *in vivo*. Nevertheless, various risk factors of diabetic cognition deficits, such as serum lipids (Kohlstedt et al., 2011), oxidative stress (Tota et al., 2012), and inflammation (Gadelha et al., 2015), could affect either the serum level or activity of ACE to a certain extent, and thus, we were unable to deduce from our data that none of these factors was significant in the early prediction of cognitive decline, except that ACE activity was related to logical memory.

Hyperglycemia and Aβ accumulation also play important roles in the memory ability of diabetic patients (Capiotti et al., 2014; Ding and Huang, 2014; Sato and Morishita, 2014). In our study, AVLT immediate recall and delayed recall tests were used to measure verbal learning and memory. The LMT test was used to measure logical memory (Gao et al., 2015), and our patients mainly exhibited logical memory disorders. Thus, this study suggests that serum ACE activity may be a more useful index for manifesting the adverse effect of hyperglycemia or Aβ accumulation on logical memory in diabetic patients. Increased ACE activity will elevate the level of angiotensin II, which will lead to oxidative stress and a reduction in cerebral blood flow that may induce memory dysfunction (Efimova et al., 2014; Nealon et al., 2016), particularly logical memory. No literature evidence is available to suggest that serum ACE level will affect memory function. Further research is necessary to elucidate the detailed mechanism and to predict the diagnosis value.

We compared the distribution of the genotype and allele frequencies of ACE I/D polymorphism between the T2DM patients with MCI and the healthy control subjects. No significant association was found between ACE I/D polymorphism and MCI in our diabetic subjects. However, our results showed that the frequency of D-allele was higher than that of I-allele in the two groups, which is consistent with Miller's research in patients with early diabetes (Miller et al., 1997). Besides, serum ACE level and activity were both significantly greater in the DD group than in the ID and II groups (*p* < 0.05) of T2DM patients with MCI, and the LMT score decreased with the increase in ACE activity. Furthermore, diabetic patients that carry the D-allele tended to develop MCI in contrast with the control group, although no statistical significance was found. Thus, we hypothesized that the D-allele was a candidate gene for memory deterioration. However, this hypothesis requires further research to be proven. In contrast to our result, previous findings showed that the D-allele was associated with reduced risk for AD (Elkins et al., 2004; Lehmann et al., 2005). Several conditions may explain these negative findings. The differences may be attributed to the small sample size and the possibility of different races, which were also reported in another study (Mathew et al., 2001). The formation of MCI may be influenced by several genes, and the ACE gene contributes only a slight effect (Vardy et al., 2012; Achouri-Rassas et al., 2016). ACE I/D polymorphism can be in a linkage disequilibrium with the true variants (McCrimmon et al., 2012). Gene–environment interactions may also lead to discrepancies (Achouri-Rassas et al., 2016).

Certain limitations of this study should be noted. The small sample, sample composition, and unknown cerebral blood flow (Steffener et al., 2013) limited the persuasion of our results to a certain degree. Moreover, ACE activity was tested via ultraviolet spectrophotometry, which is influenced by various factors, such as light source and the pH value of the solution. Long time and progressive mild visual or hearing loss are possible factors to induce cognitive deficit. We had difficulties to examine and compare the specific visual acuity/auditory ability. ACE inhibitors have been shown to inhibit the ability of ACE and prevent the formation of angiotensin II. Angiotensin receptor blockers inhibit the renin–angiotensin system by specifically blocking angiotensin II from mediating its actions through its receptors. In this study, there were no statistically significant differences in the proportion of hypertension prevalence and the use of ACEI or ARB between T2DM patients with MCI and the control groups. We roughly ignored the effects of ACEI or ARB on the results. But ACE activity might be affected by other drugs, such as calcium channel blockers (Konoshita et al., 2010), aldosterone receptor antagonist, renin inhibitors, and neural endopeptase−ACE (Regamey et al., 2002; Brown, 2003; Srinivasan et al., 2005).

Despite the aforementioned limitations, this study indicates that ACE activity can better predict memory dysfunction in diabetic patients than its serum level. The D-allele is probably a candidate gene in the memory deterioration of diabetic patients. Further studies on large population sizes are necessary to confirm this observed association and to determine whether serum ACE activity can be used as an effective biomarker for the early diagnosis of diabetic MCI.

## Trial registration

Name: Advanced Glycation End Products Induced Cognitive Impairment in Diabetes: BDNF Signal Meditated Hippocampal Neurogenesis Registration number: ChiCTR-OCC-15006060

## Author contributions

SW contributed to the idea and revised the manuscript. ST carried out the design, conduct of the study and wrote the manuscript. JH, JS, and RC carried out the data collection. RH participated in the data analysis. YS, XD, and WX helped data interpretation. All authors read and approved the final manuscript.

## Funding

This work was partially supported by the National Natural Science Foundation of China (No.81570732, SW and No. 81370921, SW).

### Conflict of interest statement

The authors declare that the research was conducted in the absence of any commercial or financial relationships that could be construed as a potential conflict of interest.
